# Synergistic effect of combination chemotherapy with praziquantel and DW-3-15 for *Schistosoma japonicum *in vitro and in vivo

**DOI:** 10.1186/s13071-021-05065-x

**Published:** 2021-10-26

**Authors:** Zi-Yin Yang, Zi-Hao Liu, Ya-Nan Zhang, Chen Li, Lei Liu, Wen-Jie Pu, Shi-Qi Xie, Jing Xu, Chao-Ming Xia

**Affiliations:** Department of Parasitology, Medical College of Soochow University, 199 Renai Road, Suzhou, 215123 China

**Keywords:** *Schistosoma japonicum*, Praziquantel, DW-3-15, Synergistic effect, Combination chemotherapy

## Abstract

**Background:**

Schistosomiasis is a debilitating and neglected tropical disease for which praziquantel (PZQ) remains the first-choice drug for treatment and control of the disease. In our previous studies, we found that the patented compound DW-3-15 (patent no. ZL201110142538.2) displayed significant and stabilized antiparasitic activity through a mechanism that might be distinct from PZQ. Here, we investigated the antischistosomal efficacy of PZQ combined with DW-3-15 against schistosomula and adult worms of *Schistosoma japonicum *in vitro and in vivo, to verify whether there was a synergistic effect of the two compounds.

**Methods:**

The antischistosomal efficacy of PZQ combined with DW-3-15 in comparison with an untreated control and monotherapy group against schistosomula and adult worms was assessed both in vitro and in vivo. Parasitological studies, scanning electron microscopy, combination index, and histopathological analysis were used for the assessment.

**Results:**

The results showed significantly reduced viability of schistosomes, achieving 100% viability reduction for juveniles and males by combination chemotherapy using PZQ together with DW-3-15 in vitro. The combination index was 0.28, 0.27, and 0.53 at the higher concentration of PZQ combined with DW-3-15 against juveniles, males, and females, respectively, indicating that the two compounds display strong synergism. Scanning electron microscopy observations also demonstrated that the compound combination induced more severe and extensive alterations to the tegument and subtegument of *S. japonicum* than those with each compound alone. In vivo, compared with the single-compound-treated group, the group treated with the higher-dose combination demonstrated the best schistosomicidal efficacy, with significantly reduced worm burden, egg burden, and granuloma count and area, which was evident against schistosomula and adult worms.

**Conclusions:**

Our study provides a potential novel chemotherapy for schistosomiasis caused by *S. japonicum*. It would improve the antischistosomal effect on schistosomula and adult worms of *S. japonicum*, and decrease individual dosages.

**Graphical Abstract:**

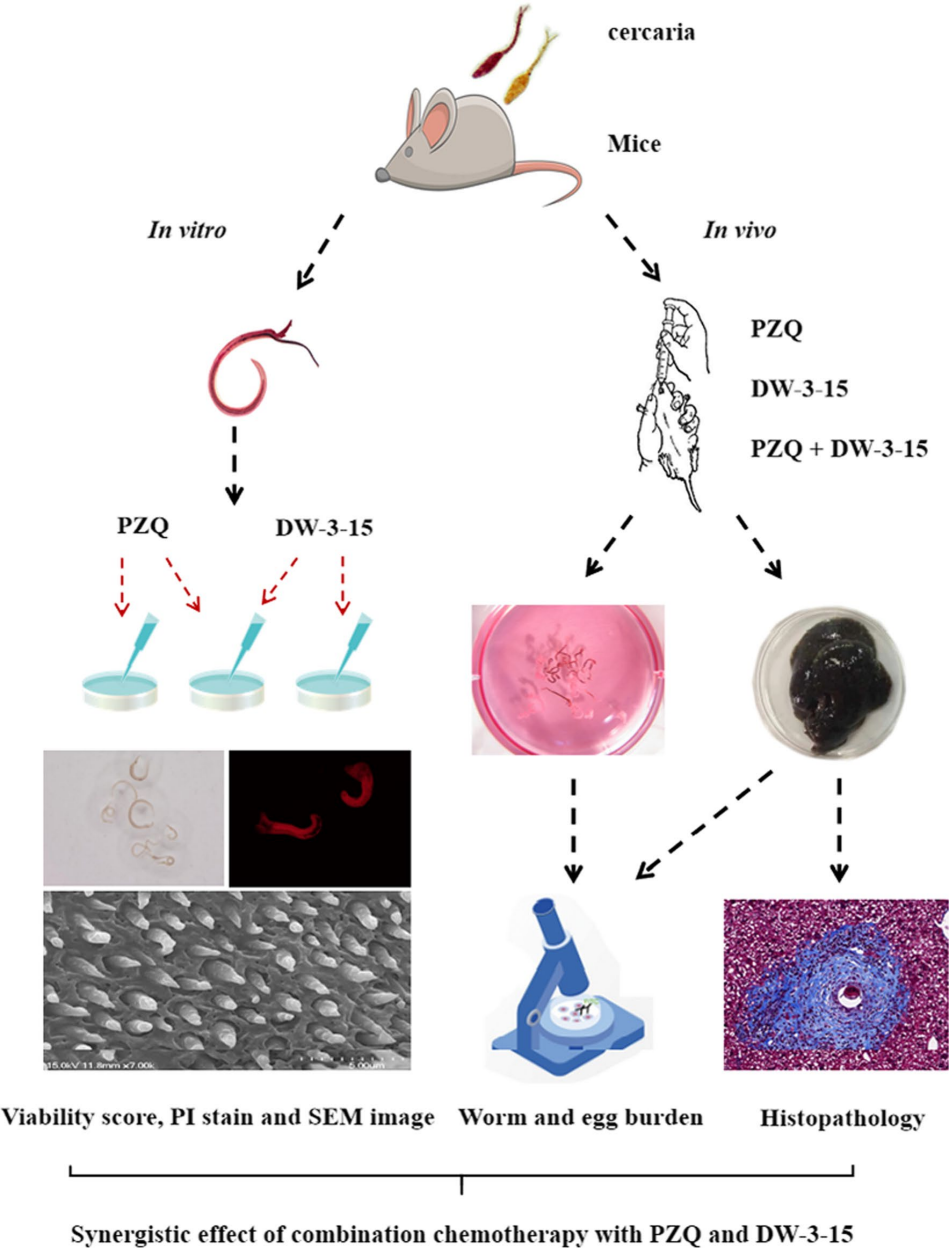

**Supplementary Information:**

The online version contains supplementary material available at 10.1186/s13071-021-05065-x.

## Background

Schistosomiasis is a major neglected tropical disease caused by *Schistosoma* trematodes, and is second only to malaria in severity among the main tropical diseases [1, 2]. More than 250 million people worldwide are infected with *Schistosoma* spp., with over 700 million at risk of infection, resulting in 1.9 million disability-adjusted life years (DALYs) per year [3, 4]. Three major species that lead to human schistosomiasis include *Schistosoma japonicum*, *S. mansoni*, and *S. haematobium* [5]. In China, only *S. japonicum* is endemic and transmitted [6]. Due to its relatively high production of eggs and serious pathological lesions, *S. japonicum* infections can be fatal if left untreated [1].

However, to date, no effective schistosome vaccine is available, and chemotherapy using praziquantel (PZQ) is still the preferred method for control and elimination of schistosomiasis [3]. PZQ, discovered at the end of the 1970s, has been recommended as the drug of first choice by the World Health Organization (WHO) for the past 40 years for the treatment of all major forms of schistosomiasis, owing to its good tolerability, low cost, broad therapeutic profile, and high efficacy with single-dose administration [7, 8]. However, whilst PZQ is effective against the adult worms of *Schistosoma* spp., it displays poor efficacy against schistosome eggs and immature schistosomula [9]. These factors may explain the low cure rates of populations in endemic areas where juveniles and adult worms coexist. Furthermore, due to the repeated long-term and large-scale treatment of PZQ for schistosomiasis, the increasing appearance of drug resistance is cause for great concern. In 1994, the first study performed by Fallon and Doenhoff demonstrated that it was possible to induce *S. mansoni* less susceptible to PZQ in a mouse model [10]. Thereafter, the emergence of resistance to PZQ for treatment of schistosomiasis has been reported both clinically and in the laboratory, highlighting the urgent need for alternative drugs against schistosomiasis [11].

Drug repurposing may offer a low-risk and cost-effective solution in the search for novel antischistosomal drugs. Artemisinin analogs such as artemether and artesunate, originally discovered as antimalarial drugs, have promising activity against the young developmental stages of schistosomes [12]. Based on the complementary activity of PZQ and artemisinin, a novel compound linked to the endoperoxide bridge of artemisinin at position 10 of PZQ, termed DW-3-15 (patent no. ZL201110142538.2), was developed in our laboratory [13]. DW-3-15 has shown outstanding antischistosomal properties against all developmental stages of *S. japonicum*. Notably, compared with PZQ, DW-3-15 showed improved antiparasitic activity against juvenile worms, while still demonstrating promising schistosomicidal efficacy against adults [13]. In addition, the commercial DW-3-15 synthesized by a pharmaceutical company showed reproducible antischistosomal activity, highlighting its activity and stability [14]. Although DW-3-15 was highly effective against schistosomes, with worm reduction rates ranging from 63.4 to 85.7%, it failed to clear all the worms from *S. japonicum*-infected mice [13, 14]. Similar observations were reported for PZQ, which failed to achieve 100% worm reduction when used alone [15].

Considering the advantages of increased efficacy, decreased toxicity, and reduced risk of drug resistance in chemotherapy, drug combination may provide an alternative strategy for disease treatment [12, 16–18]. In this study, we investigated the schistosomicidal activity of the combination of PZQ and DW-3-15 against multiple developmental stages of *S. japonicum *in vitro and in vivo. We also explored the synergistic effect of combination chemotherapy with PZQ and DW-3-15 against *S. japonicum.*

## Methods

### Ethics statement

All the animal experiments in the present study were carried out in strict accordance with the Regulations for the Care and Use of Laboratory Animals of the National Institutes of Health. The protocol was approved by the committee on the ethics of animal experiments of Soochow University (permit number: 2007–13).

### Parasites and animals

*Schistosoma japonicum*-infected snails (*Oncomelania hupensis*) were provided by the National Institute of Parasitic Diseases, Chinese Center for Disease Control and Prevention (Shanghai, China). *Schistosoma japonicum* cercariae (Chinese mainland strain) shed from the snails were used to infect mice. Female Institute of Cancer Research (ICR) mice, aged 4–6 weeks and weighing 15–25 g, were provided by the Experimental Animal Center of Soochow University (Suzhou, China). All mice were raised under specific pathogen-free conditions with controlled temperature (20 ± 2 °C) and photoperiod (12 h light, 12 h dark). Each mouse was transcutaneously infected with 60 ± 5 *S. japonicum* cercariae.

### Reagents

PZQ derivative DW-3-15 (patent no. ZL201110142538.2) was synthesized by WuXi AppTec Co., Ltd, (Shanghai, China). The synthetic route and chemical characterization data for DW-3-15 have been described previously [14]. PZQ was purchased from Sigma-Aldrich (St. Louis, MO, USA). For in vitro assay, all chemicals were dissolved in dimethyl sulfoxide (DMSO, Fluka, Buchs, Switzerland). For the in vivo test*,* PZQ and the commercial DW-3-15 powder were dissolved in a solution of 0.5% carboxymethyl cellulose sodium (Sigma-Aldrich, St. Louis, MO, USA). Suspension of the two compounds was administered intragastrically to mice. Dulbecco’s modified Eagle’s medium (DMEM) and penicillin/streptomycin were purchased from Life Technologies (Carlsbad, CA, USA). Newborn calf serum was provided by Biological Industries (Cromwell, CT, USA).

### In vitro treatment

*Schistosoma japonicum* worms recovered from infected mice at 14 days post-infection (juvenile worm) and 35 days post-infection (adult worm) by perfusion of the hepatic portal system and mesenteric veins [19] were placed in six-well plates (Corning Costar, Corning, NY, USA) containing complete DMEM supplemented with 10% newborn calf serum, 100 μg/ml streptomycin, and 100 U/ml of penicillin, and incubated at 37 °C in a 5% CO_2_ incubator. Juvenile worms were divided into four groups, with five worms per well, each being tested in triplicate, as follows: group I, untreated control, incubated with complete DMEM containing 0.1% DMSO; group II, worms treated with PZQ; group III, worms treated with DW-3-15; group IV, worms treated with a combination of PZQ and DW-3-15. Groups II and III were further subdivided into four subgroups (groups IIa–IId and groups IIIa–IIId, respectively) to test the efficacy of different concentrations of PZQ and DW-3-15 (25, 50, 75, and 100 μM). Worms in group IV were subdivided into two subgroups to test different combinations of PZQ and DW-3-15. Worms in group IVa were treated with PD^a^ (50 μM PZQ combined with 50 μM DW-3-15), and worms in group IVb were treated with PD^b^ (100 μM PZQ combined with 100 μM DW-3-15). Adult worms separated by sex were also divided into four group, with five worms per well and with the same treatment schedule as juvenile worms. All the *S. japonicum* worms were exposed to the chemicals for 16 h, rinsed three times with DMEM the next day, then cultured in chemical-free complete DMEM for 72 h. At 24, 48, and 72 h post-incubation, worm status was checked under a dissecting microscope (SZX16, Olympus, Japan), and viability was scored as described previously [20], based on phenotypical changes including mobility and general appearance. Briefly, the viability scores ranged from 0 (severely compromised) to 3 (no effect). The following phenotype scoring criteria were used: 3 = worm moves actively and has a transparent body; 2 = worm moves stiffly and slowly, and has a translucent body; 1 = worm moves partially and turns opaque; 0 = dead [20]. For each sample the following formula was used: viability score = ∑(worm scores)/number of worms. Each experiment was carried out at least twice.

### Worm viability by fluorescence microscopy

Worm viability was further confirmed by propidium iodide (PI) fluorescence assay as described previously [21, 22]. Briefly, PI was added to each well to obtain a final concentration of 2.0 mg/ml, and incubated with worms for 15 min at 37 °C. Then, worms were observed by an inverted fluorescence microscope (Eclipse TS100, Nikon, Japan) to capture color bright-field and fluorescence red channel (586 nm) images.

### Scanning electron microscopy

Ultrastructural features of the schistosome worms treated with the combination of PZQ and DW-3-15 (groups IVa and IVb) were examined using scanning electron microscopy (SEM) and were compared with those of the untreated control group (group I) and the monotherapy groups (groups IId and IIId). For SEM, worms were washed three times in phosphate-buffered saline (PBS; pH 7.4) and fixed overnight at 4 °C in 2.5% glutaraldehyde-PBS solution (pH 7.4). Then the worms were washed again in PBS, post-fixed in 1% osmium tetroxide (OsO4) for 1 h, and dehydrated in an ascending series of ethanol. Finally, the samples were dried, mounted on aluminum stubs, coated with gold, and examined using a Hitachi S-4700 scanning electron microscope (Chiyodaku, Japan).

### In vivo treatment

Female 4- to 6-week-old ICR mice were transcutaneously infected with 60 ± 5 *S. japonicum* cercariae. The infected mice were randomly divided into four groups. Group I, infected and untreated (*n* = 5), received vehicle (0.5% carboxymethyl cellulose sodium) only. For group II, infected and treated with PZQ, in order to evaluate single chemical efficacy, this group was subdivided into three subgroups (groups IIA–IIC) of five mice each, which received a single dose of 100, 200, or 400 mg/kg per day orally for five consecutive days. Group III, infected and treated with DW-3-15, was similarly subdivided into three subgroups (groups IIIA–IIIC) of five mice each, which received an orally administered single dose of 100, 200, or 400 mg/kg DW-3-15. Group IV, infected and treated with the combination of PZQ and DW-3-15, was subdivided into two subgroups (groups IVA and IVB) of five mice each, which received PD^c^ (100 mg/kg PZQ combined with 200 mg/kg DW-3-15) and PD^d^ (200 mg/kg PZQ combined with 400 mg/kg DW-3-15), respectively. To study the stage-specific susceptibility to the chemicals, treated groups II, III, and IV were subsequently subdivided according to the onset day of treatment. Subgroups IIA-a, IIB-a, IIC-a, IIIA-a, IIIB-a, IIIC-a, IVA-a, and IVB-a were treated at 14 days post-infection (targeting the juvenile stage) for five consecutive days. Subgroups IIA-b, IIB-b, IIC-b, IIIA-b, IIIB-b, IIIC-b, IVA-b, and IVB-b were treated at 28 days post-infection (targeting the adult worm stage) for five consecutive days. To investigate the individual/combination efficacy of PZQ and DW-3-15 against juvenile and adult schistosomes harbored in the same host, mice of subgroups IIA-c, IIB-c, IIC-c, IIIA-c, IIIB-c, IIIC-c, IVA-c, and IVB-c were initially infected with 30 schistosome cercariae, followed by a second infection with the same number of cercariae 14 days after the first infection. These mice were then treated on day 7 after the second infection for five consecutive days. All the experiments were carried out in triplicate.

### Identification of worm burden and egg burden

At 21 days post-treatment, all mice were perfused using a method described previously [19], and the recovered worms separated by sex were counted. Mouse livers of fixed weight were incubated overnight in 5% sodium hydroxide (NaOH) at 65 °C for 1 h, and the mean number of eggs per gram of tissue was then calculated. The percentage reduction in worm burden and egg burden was calculated using the following formula: reduction rate (%) = [(mean value of control group − mean value of treated group)/mean value of control group] × 100%.

### Histopathological assessment

Mouse livers from all groups were washed with PBS (pH 7.4) and fixed in 10% neutral buffered formalin. Fixed livers were dehydrated in increasing concentrations of ethanol, diaphonized in xylol, and then embedded in paraffin. Sections 4 μm thick were stained with hematoxylin and eosin (H&E) for granuloma analysis. The areas of granuloma were calculated at ×20 magnification.

### Combination index

Combination index (CI) analysis was employed to evaluate the synergistic effects of the combination of PZQ and DW-3-15, where CI > 0.1 indicates very strong synergism, CI 0.1–0.3 strong synergism, CI 0.3–0.7 synergism, CI 0.7–0.85 moderate synergism, CI 0.85–0.9 slight synergism, CI 0.9–1.1 nearly additive, and CI > 1.1 antagonism, as described previously [23, 24]. CI was calculated using CompuSyn v.1.0 software (ComboSyn, Inc., Paramus, NJ, USA).

### Statistical analysis

The results of the study were expressed as standard error of the mean (SEM) using IBM SPSS Statistics version 23 software. All statistical analyses were performed using GraphPad Prism version 7.0 software. Differences between the treated and the control groups were compared by one-way analysis of variance (ANOVA) followed by Dunnett’s test. A *P*-value < 0.05 was considered to be statistically significant.

## Results

### Effects of individual compounds on the viability of *S. japonicum* in vitro

In order to select the optimal concentration of PZQ and DW-3-15 for combination therapy, the antischistosomal activity of PZQ and DW-3-15 individually was assayed at concentrations of 25–100 μM. As shown in Additional file 1: Tables S1–S3, with ascending concentrations of PZQ, the viability of juveniles, males, and females was significantly reduced compared with the control group (juveniles: *F*_(10, 319)_ = 56.97, *P* < 0.0001; males: *F*_(10, 319)_ = 98.59, *P* < 0.0001; females: *F*_(10, 319)_ = 90.66, *P* < 0.0001), with PZQ at 100 μM showing the highest viability reduction rate in juveniles, males, and females. However, as the incubation period continued, juvenile, male, and female worms gradually recovered after 48 h exposure to PZQ, in accord with our previous study [14]. For DW-3-15, unlike PZQ, the antischistosomal effect was both concentration- and time-dependent, with DW-3-15 at 100 μM demonstrating the most potent effect. However, neither compound was capable of killing all the worms. In order to investigate whether combination therapy could enhance the antischistosomal performance of the two compounds, juveniles, males, and females were incubated with a constant dose ratio (1:1) of the lower concentration (50 μM, PD^a^) and the higher concentration (100 μM, PD^b^) of PZQ combined with DW-3-15.

### The combination compounds significantly reduced the viability of *S. japonicum* in vitro

**Fig. 1:**
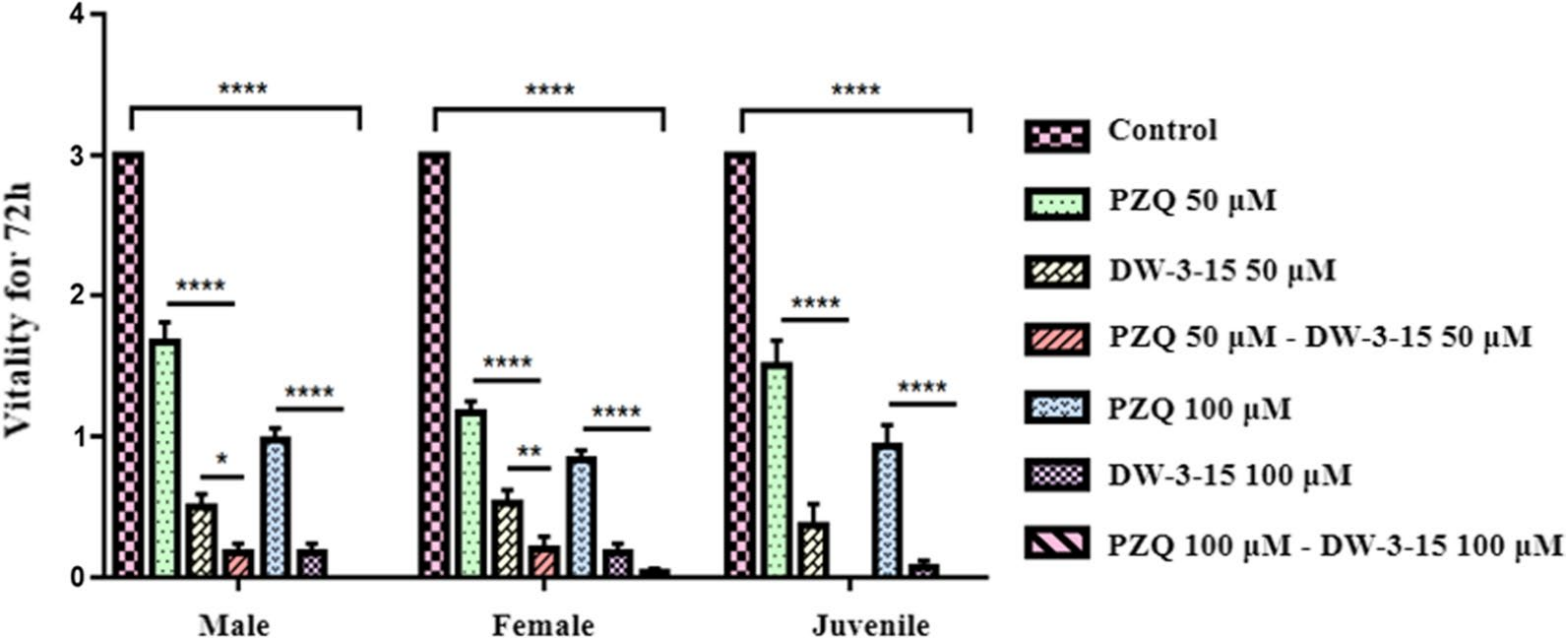
The changes in the viability of *S. japonicum* adult and juvenile worms in vitro. Adult and juvenile worms were exposed for 72 h to PZQ, DW-3-15, and their combinations at different concentrations in vitro. The viability was evaluated using a viability score of 0–3. The control group was incubated with complete DMEM with 0.1% DMSO. Data represent the mean ± SEM from multiple-group experiments. Statistical analysis was performed among control, single-compound, and combination groups using one-way ANOVA followed by Dunnett’s test. Significant differences are indicated by *P < 0.05, **P < 0.01, and ****P < 0.0001

As shown in Fig. 1, there was a statistically significant difference in terms of viability among the different groups against juveniles (*F*_(6, 203)_ = 109.3, *P* < 0.0001), males (*F*_(6, 203)_ = 181.2, *P* < 0.0001), and females (*F*_(6, 203)_ = 217.8, *P* < 0.0001). In comparison with the PZQ monotherapy group, a combination of PZQ and DW-3-15 at different concentrations induced a more significant reduction in viability of juveniles (PD^a^: *F*_(6, 203)_ = 109.3, *P* < 0.0001; PD^b^: *F*_(6, 203)_ = 109.3, *P* < 0.0001), males (PD^a^: *F*_(6, 203)_ = 181.2, *P* < 0.0001; PD^b^: *F*_(6, 203)_ = 181.2, *P* < 0.0001), and females (PD^a^: *F*_(6, 203)_ = 217.8, *P* < 0.0001; PD^b^: *F*_(6, 203)_ = 217.8, *P* < 0.0001). There was also a more significant reduction in viability of males (PD^a^: *F*_(6, 203)_ = 181.2, *P* = 0.0189 ) and females (PD^a^: *F*_(6, 203)_ = 217.8, *P* = 0.0053) treated with the combination of PZQ and DW-3-15 compared with DW-3-15 monotherapy. Notably, exposure to the higher-concentration combination resulted in 100% reduction in viability of juvenile and male adult worms after 72 h (Additional file 1: Tables S1–S3), indicating that the combination of the two compounds enhanced the anthelmintic performance of the individual compounds. No significant difference in viability was observed between the two concentrations of the combinations (Fig. 1).

**Fig. 2:**
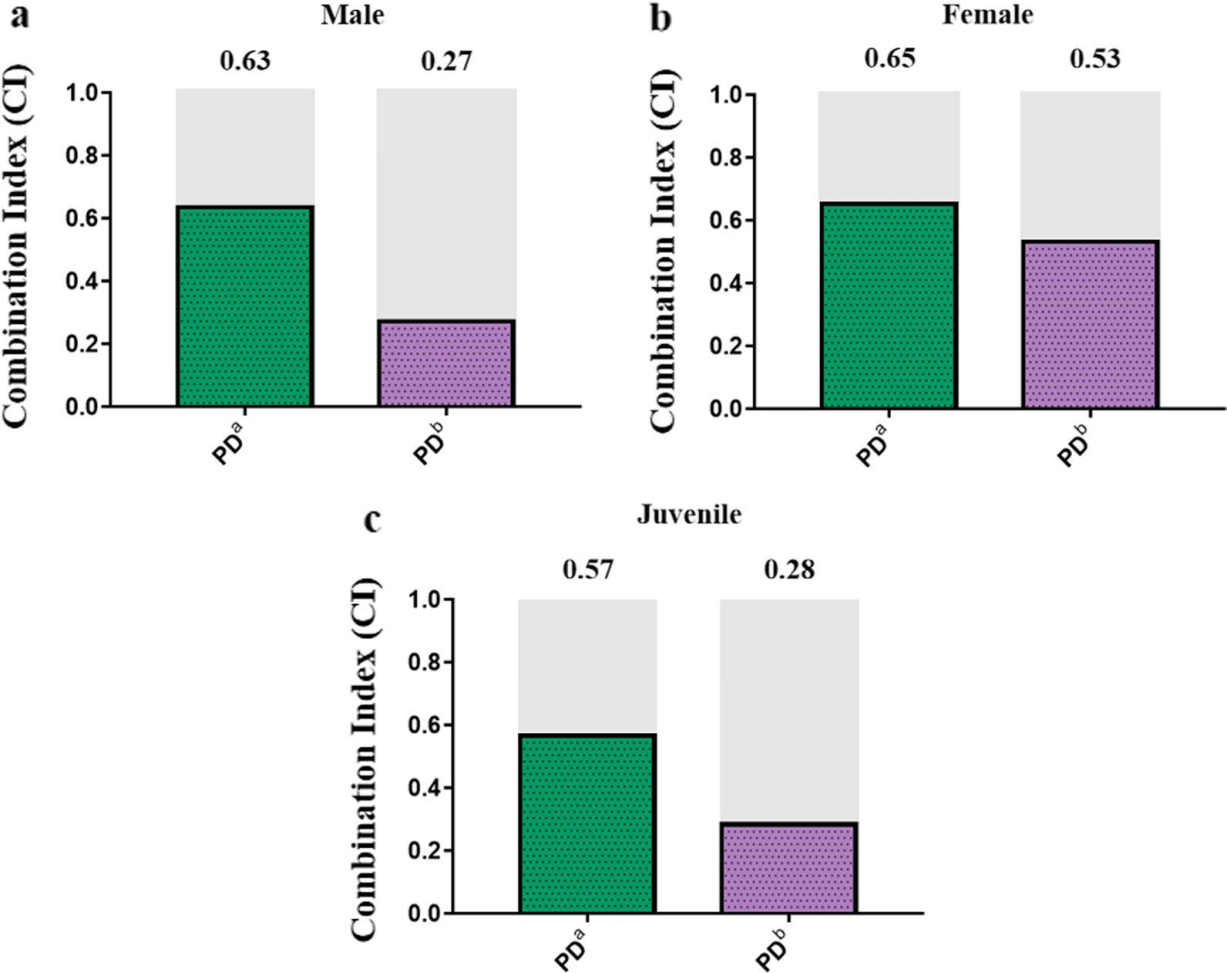
Combination indices (CI) of two compounds against adults and juveniles of *S. japonicum *in vitro. CI were obtained from the combination of PZQ and DW-3-15 against male (**a**), female (**b**), and juvenile (**c**) worms of *S. japonicum* after 72 h of incubation in vitro. Synergism is marked in gray. PD^a^ = 50 μM PZQ combined with 50 μM DW-3-15; PD^b^ = 100 μM PZQ combined with 100 μM DW-3-15

**Fig. 3:**
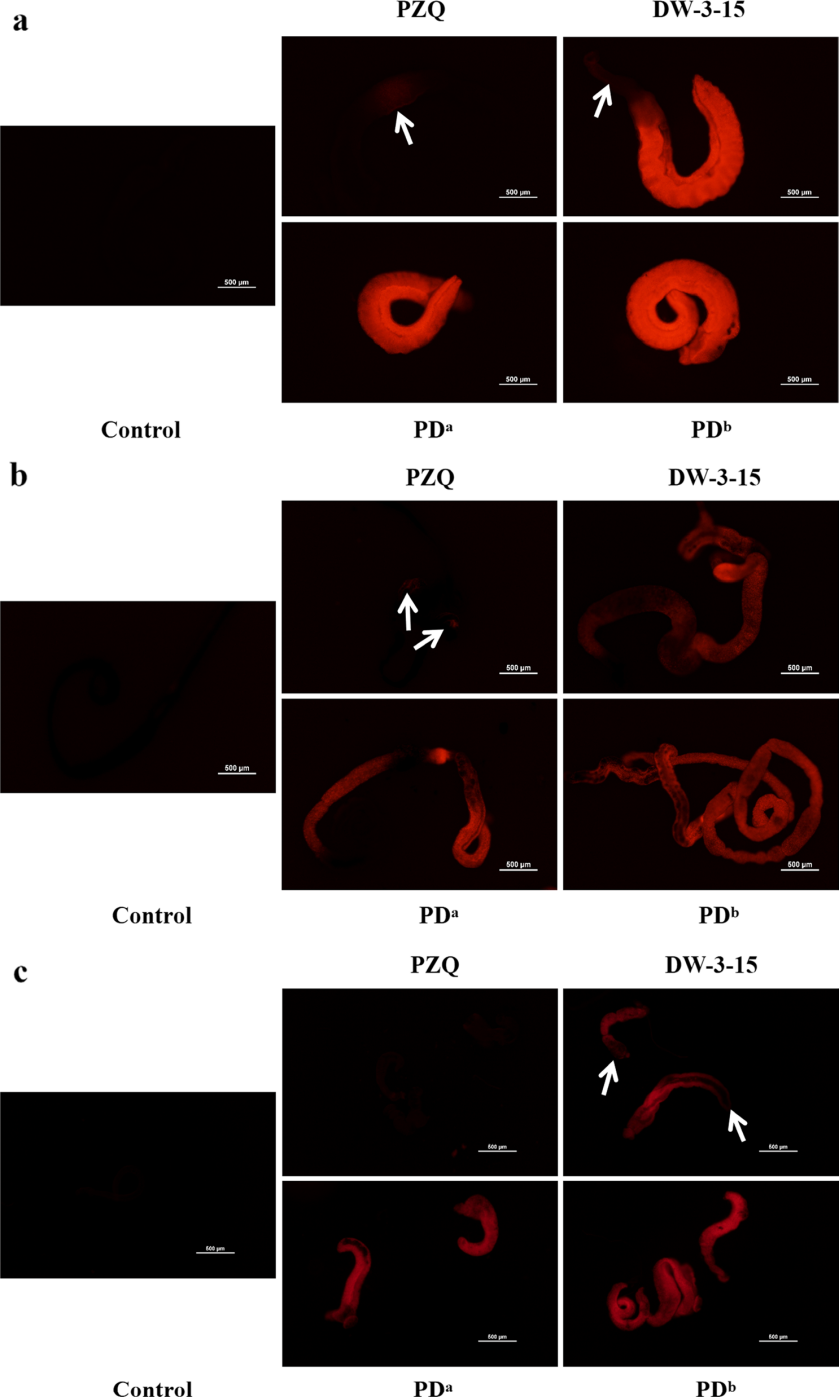
Antischistosomal effects of PZQ, DW-3-15, and their combinations in vitro*.* The viability of *S. japonicum* males (**a**), females (**b**), and juveniles (**c**) was assessed by propidium iodide (PI) incorporation following exposure for 72 h. The worms were exposed to 100 μM PZQ, 100 μM DW-3-15, PD^a^ (50 μM PZQ combined with 50 μM DW-3-15), and PD^b^ (100 μM PZQ combined with 100 μM DW-3-15) for 16 h, washed three times with DMEM the next day, and then cultured in drug-free complete DMEM for 72 h. The untreated control group was incubated in complete DMEM with 0.1% DMSO. The worms were then treated with PI for 15 min at 37 °C. Notably, stronger fluorescence signals were observed in worms exposed to DW-3-15 and the combination compounds than to PZQ alone, indicating that the tissue of the former was markedly damaged. Scale bars: 500 μm

The CI of the lower-concentration combination of PZQ and DW-3-15 (PD^a^) against juveniles, males, and females was 0.57, 0.63, and 0.65, respectively; for the higher-concentration combination group (PD^b^), the CI was 0.28, 0.27, and 0.53, respectively (Fig. 2a–c). Based on the results, we concluded that the combination of the two compounds induced a synergistic effect against *S. japonicum*, and the higher-concentration combination group showed stronger synergistic effects in males and juveniles. In addition to the bright-field microscopic assessment, the viability of the worms was assessed by a red-fluorescent dye propidium iodide (PI) that objectively detects dead parasites during in vitro culture. Following 72 h incubation with individual or combined PZQ and DW-3-15 at different concentrations, dead worms were stained with PI showing red fluorescence signals. As expected, worms treated with combination compounds (PD^a^ and PD^b^) displayed the brightest red fluorescence intensity among juveniles, males, and females (Fig. 3a–c). The fluorescence intensity of the worms in the PZQ group was similar to that of the untreated control groups (Fig. 3a–c). In addition, worms incubated with DW-3-15 alone were also stained by PI, and the fluorescence intensity was stronger than in worms with PZQ monotherapy (Fig. 3a–c).

### Morphological analysis by SEM

**Fig. 4:**
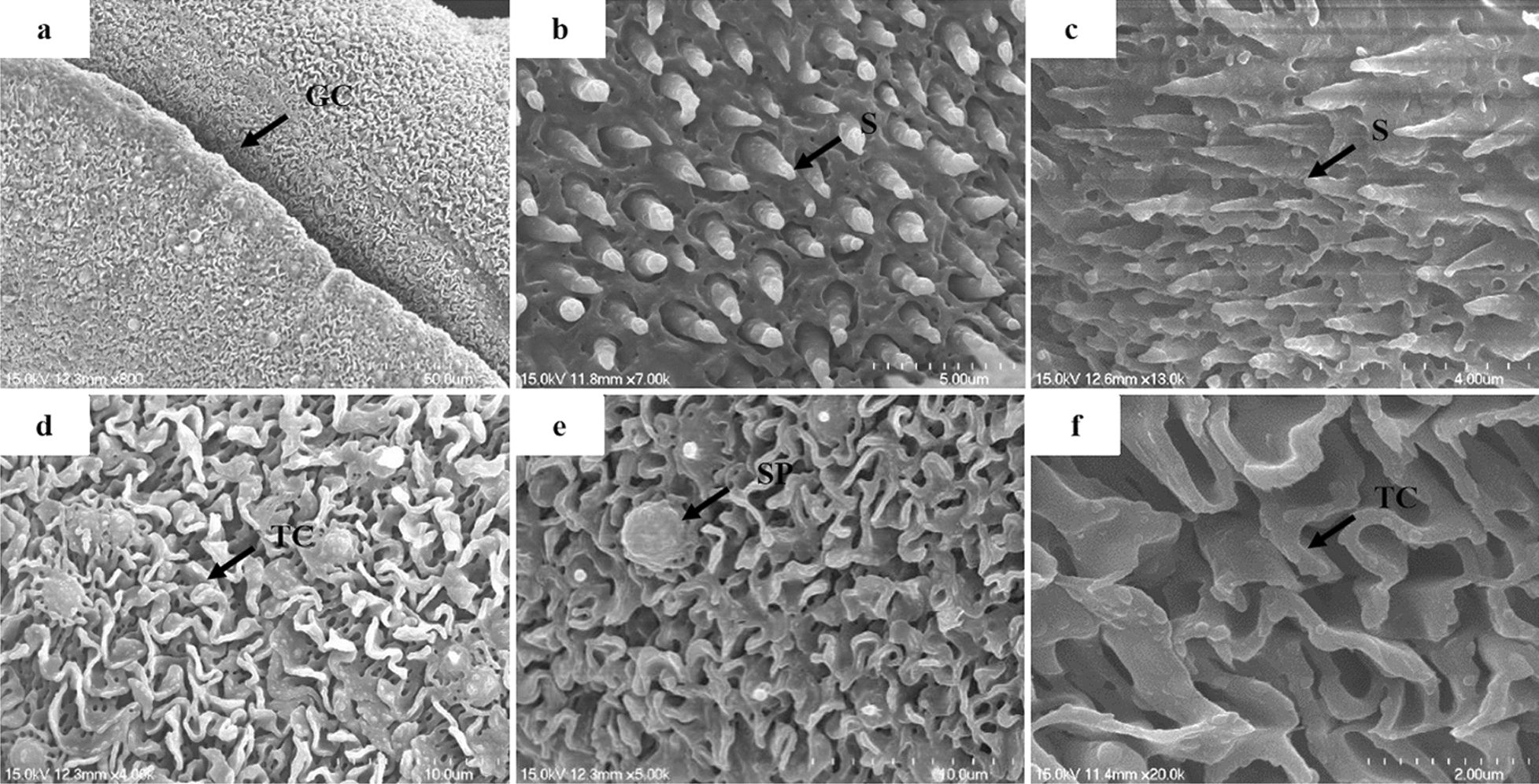
Scanning electron micrographs of *S. japonicum* males of the negative control group. Worms after 72 h of incubation showed no abnormalities in tegument topography or the gynecophoral canal (GC) (**a**). Notably, the morphology of the oral and ventral suckers was normal, showing high integrity and prominent spines (S) (**b**, **c**). Numerous tegumental crests (TC) with sensory papillae (SP) appeared along the body (**d**–**f**). Scale bars: **a**, 50 µm; **b**, 5 µm; **c**, 4 µm; **d**, 10 µm, **e**, 10 µm, **f**, 2 µm

Males from the control group showed normal ultrastructural features (Fig. 4). The oral and ventral suckers and gynecophoral canals showed no obvious morphological alterations, with apically directed spines in the suckers (Fig. 4a–c). The tegument of the mid-body showed numerous crests with sensory papillae that were distributed along the body (Fig. 4d–f).

Males exposed to 100 μM PZQ showed various changes in the tegument and gynecophoral canal, including extensive blebs, shallow peeling, pit-shaped erosion, and the rupture of the crests (Additional file 2: Figure S1a, d–f). In the oral and ventral suckers, disarrangement of the inner wall and a loss of spines were observed (Additional file 2: Figure S1b, c). Alterations in the tegument caused by 100 μM DW-3-15 were more significant than those from 100 μM PZQ. Along the gynecophoral canal and tegument of worms, sensory papillae exhibited severe damage, with the tegumental structures showing marked lysis, disorganization, and collapse, with pronounced blisters and hole-shaped erosions (Additional file 3: Figure S2a, d–f). Fusion of the inner wall and a loss of spine structure were observed in the oral and ventral suckers of males, with hole-shaped formations evident in the oral suckers (Additional file 3: Figure S2b, c). It was noteworthy that changes in the worms in the PZQ group were confined to the tegument, with no damage observed in the muscle tissue layer (Additional file 2: Figure S1). In contrast, DW-3-15 induced the exposure of the subtegument layer of the muscle tissue in some regions (Additional file 3: Figure S2).

**Fig. 5:**
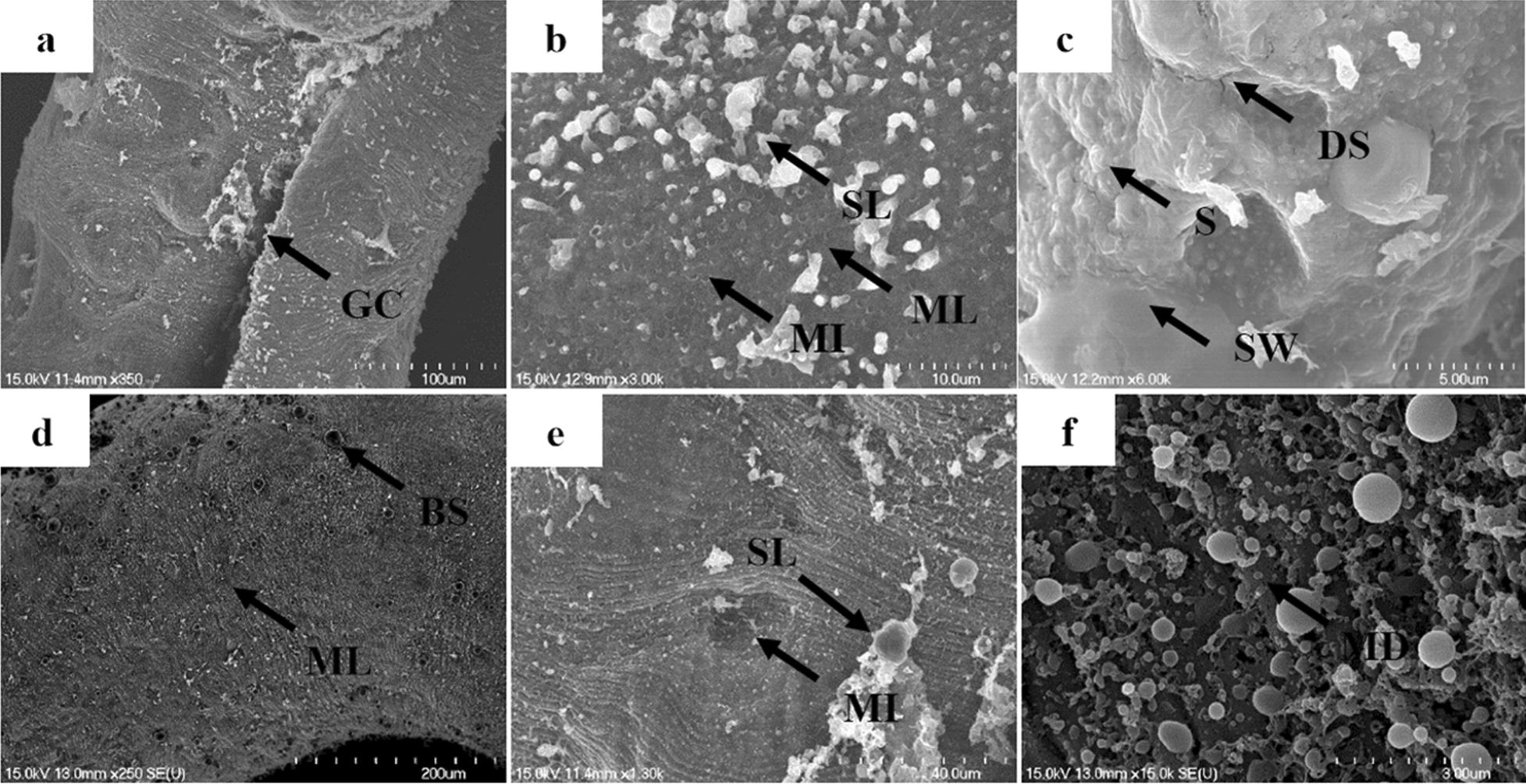
Scanning electron micrographs of *S. japonicum* male worms exposed to PD^b^. After 72 h of incubation, the gynecophoral canal (GC), oral sucker, and tegumental crest showed severe injury including blisters (BS) and extensive sloughing (SL), with exposure of the subtegumental muscle layer (ML), muscle injury (MI), and muscle dissolution (MD) (**a**, **b**, **d**–**f**). The ventral sucker of the worms showed extensive swelling (SW), surface fusion, and spine loss (S), with the formation of long irregular disorganized splits (DS) (**c**). PD^b^ = 100 µM PZQ combined with 100 µM DW-3-15. Scale bars: **a**, 100 µm; **b**, 10 µm; **c**, 5 µm; **d**, 200 µm; **e**, 40 µm; **f**, 3 µm

When the two compounds were combined, extensive tegumental damage appeared along the whole tegument and in the gynecophoral canal (Additional file 4: Figure S3; Fig. 5). PD^a^-treated males showed severe tegumental damage in the form of hole-shaped erosions, blisters, and extensive sloughing, causing exposure of the subtegumental muscle layer and muscle injury (Additional file 4: Figure S3a, d–f). Images showing tegument swelling of the oral and ventral suckers with focal areas of disintegration and fusion are shown in Additional file 4: Figure S3b and c. More significant tegumental changes and sucker deformity were observed in worms exposed to PD^b^. Extensive sloughing of the tegument, with almost complete subtegumental muscle layer exposure, blisters, muscle injury, and muscle dissolution were observed in the whole worm tegument and gynecophoral canal (Fig. 5a, d–f). All the subtegumental structures of the oral suckers were exposed, with hole-shaped erosions on the muscle layer (Fig. 5b). The ventral sucker showed extensive swelling and collapse at the tegument, spine loss, and complete fusion, and long, irregular disorganized splits appeared (Fig. 5c).

### Significant reduction in worm burden by combination chemotherapy in vivo

**Fig. 6:**
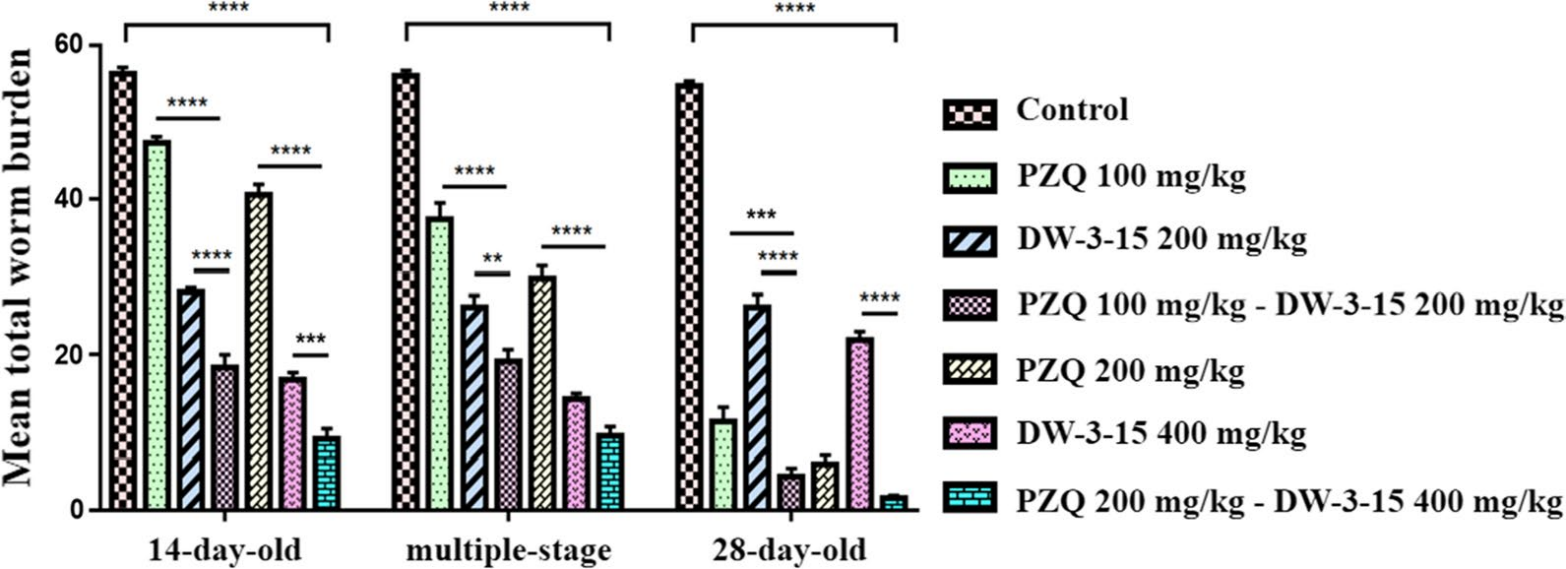
Antischistosomal effect of PZQ combined with DW-3-15 in vivo. The untreated control group was treated with 0.5% carboxymethyl cellulose sodium. Compounds were given to mice at 14 days post-infection (against 14-day-old juvenile), 21 days post-infection (with a reinfection at 14 days post-infection, against multiple developmental stages), and 28 days post-infection (against 28-day-old adult worms). Data represent mean ± SEM from multiple-group experiments. Statistical analysis was performed among control, monotherapy, and combination groups using one-way ANOVA followed by Dunnett’s test. Significant differences are indicated by **P < 0.01, ***P < 0.001, and ****P < 0.0001

**Fig. 7:**
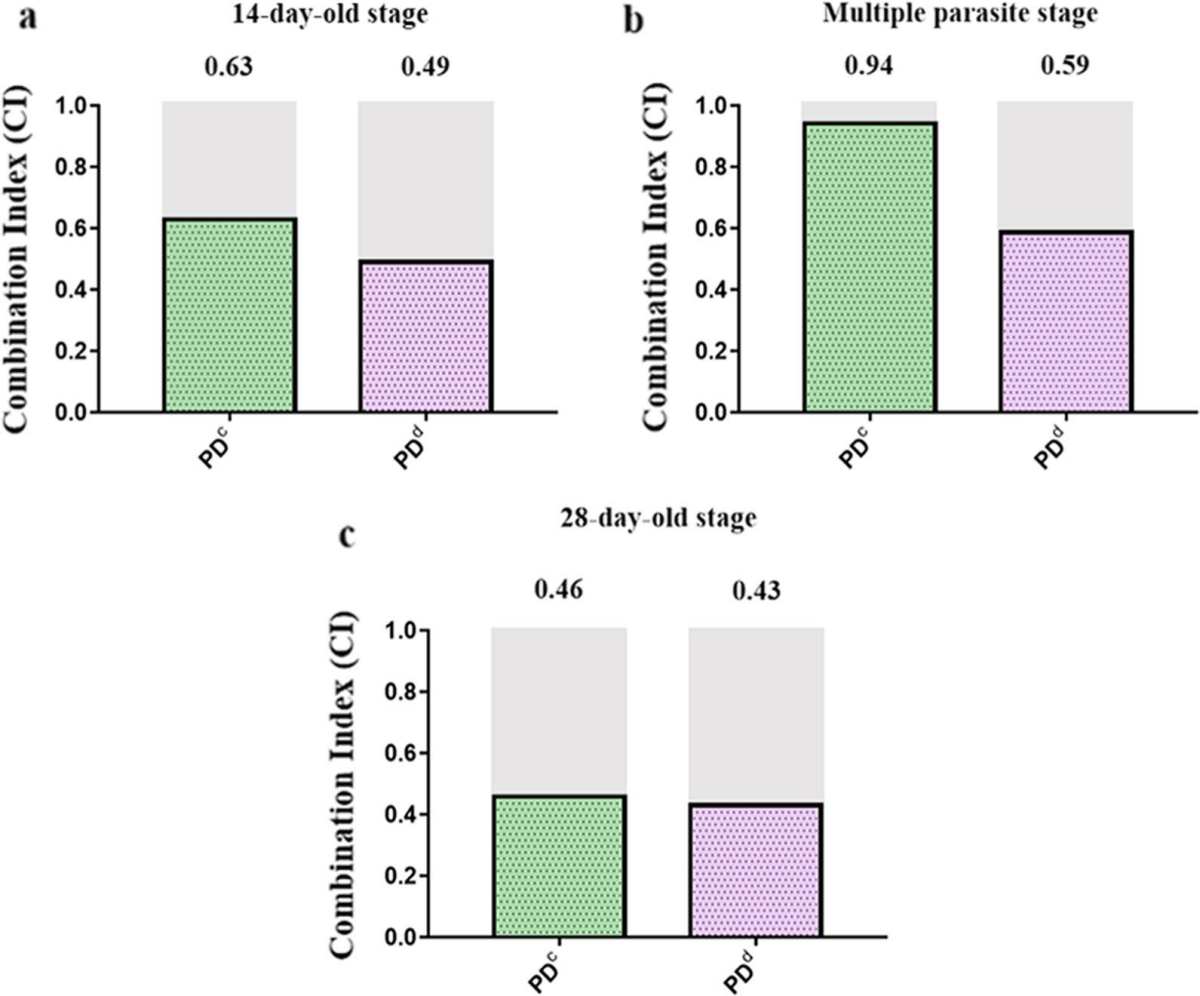
Combination indices (CI) of PZQ combined with DW-3-15 against *S. japonicum *in vitro. CI were obtained from combinations of PZQ and DW-3-15 against 14-day-old (**a**), multiple-stage (**b**), and 28-day-old worms (**c**) of *S. japonicum* in vivo. Synergism is indicated in gray. PD^c^ = 100 mg/kg PZQ combined with 200 mg/kg DW-3-15; PD^d^ = 200 mg/kg PZQ combined with 400 mg/kg DW-3-15

Data for worm and egg burden assessment in the study subgroups are shown in Fig. 6 and in Additional file 1: Tables S4–S6. Oral administration of the two-dose combination, PD^c^ (100 mg/kg PZQ combined with 200 mg/kg DW-3-15) and PD^d^ (200 mg/kg PZQ combined with 400 mg/kg DW-3-15), to infected mice against 14-day-old juvenile, 28-day-old adult, and multiple parasitic stages resulted in a statistically more significant reduction in the mean total worm burden (Fig. 6; juveniles: *F*_(6, 98)_ = 192.6, *P* < 0.0001; adults: *F*_(6, 98)_ = 229.1, *P* < 0.0001; multiple parasitic stages: *F*_(6, 98)_ = 123.1, *P* < 0.0001) compared with the infected but untreated control group. Mice treated with a higher-dose combination (PD^d^) against 14-day-old juveniles, 28-day-old adults, and multiple parasitic stages showed the highest reductions in total worm burden, at 83.8, 97.3, and 83.0%, respectively (Additional file 1: Tables S4–S6). Respective reductions in total worm burden induced in the corresponding PD^c^ group against 14-day-old juveniles, 28-day-old adults, and multiple parasitic stages were 67.5, 92.1, and 65.9% (Additional file 1: Tables S4–S6). As expected, PZQ monotherapy was effective against adult worms but was less effective against juveniles. Reductions induced by PZQ monotherapy at the highest dose (400 mg/kg) against juvenile, adult, and multiple-stage worms was 43.3, 96.9 and 64.1%, respectively (Additional file 1: Tables S4–S6). For the DW-3-15 monotherapy group, the efficacy against juvenile, adult, and multiple-stage worms was dose-dependent, with increasing dose resulting in a more significant decrease in total worm burden. Reductions induced by DW-3-15 against the corresponding developmental stages at the highest dose (400 mg/kg) were 70.3, 60.0, and 74.5%, respectively (Additional file 1: Tables S4–S6). Compared with PZQ monotherapy, a statistically significant reduction in total worm burden was observed for treatment with the two-compound combinations against juveniles (Fig. 6; *F*_(6, 98)_ = 192.6, *P* < 0.0001) and multiple parasitic stages (Fig. 6; *F*_(6, 98)_ = 123.1, *P* < 0.0001). Although there was no significant difference in the reduction of total worm burden between the higher-dose combination compounds and PZQ alone at 200 mg/kg against adults, the combination groups displayed a higher reduction than PZQ alone. Compared with DW-3-15 monotherapy, a statistically significant reduction in total worm burden was found with combination-group treatment against juveniles and adult worms (Fig. 6; juveniles: *F*_(6, 98)_ = 192.6, *P* = 0.0001; adults: *F*_(6, 98)_ = 229.1, *P* < 0.0001). With regard to the efficacy against multiple parasitic stages harbored in one host, although there was no statistically significant difference between combination therapy and DW-3-15 monotherapy at the highest dose, the reduction in total worm burden was higher using combination therapy than DW-3-15 monotherapy (Fig. 6). The results indicated that the PZQ and DW-3-15 combined application was synergistically active against multiple developmental stages of *S. japonicum*. Synergism was observed for a higher-dose combination of PZQ and DW-3-15 (PD^d^) against 14-day-old juveniles, 28-day-old adult worms, and multiple-parasitic-stage worms. The CI was 0.49, 0.43, and 0.59, respectively (Fig. 7).

### Significant decrease in hepatic granulomas by combination chemotherapy

The histopathological features of granulomas in Masson trichrome-stained mouse liver sections are shown in Additional file 5: Figure S4. The livers of untreated infected mice showed typical hepatic schistosomal granulomas, surrounded by large numbers of inflammatory cells. Pronounced reductions in *S. japonicum*-associated hepatic granuloma size were observed in all developmental stages in infected mice treated with PZQ and DW-3-15 combinations (Additional file 5: Figure S4; juveniles: PD^c^: *F*_(4, 10)_ = 11.5, *P* = 0.0018, PD^d^: *F*_(4, 10)_ = 11.5, *P* = 0.0010; multiple parasitic stages: PD^c^: *F*_(4, 10)_ = 14.9, *P* = 0.0011, PD^d^: *F*_(4, 10)_ = 14.9, *P* = 0.0002; adults: PD^c^: *F*_(4, 10)_ = 9.731, *P* = 0.0027, PD^d^: *F*_(4, 10)_ = 9.731, *P* = 0.0007). By calculating the egg burden per gram of liver tissue, all treatments were found to induce statistically significant reductions in liver egg burden when compared with the infected untreated control (juveniles: *F*_(8, 81)_ = 184.6, *P* < 0.0001; multiple parasitic stages: *F*_(8, 83)_ = 114.8, *P* < 0.0001; adults: *F*_(8, 82)_ = 134.8, *P* < 0.0001). The group treated with the higher-dose combination of PZQ and DW-3-15 (PD^d^) showed the greatest decrease in egg burden (Additional file 1: Tables S4–S6).

## Discussion

Human schistosomiasis is an important neglected disease in tropical and subtropical areas [25]. PZQ is the first-choice drug available for the treatment of schistosomiasis and has been used extensively for mass administration and transmission control in the absence of alternative drugs [26]. However, the long-term and mass use of PZQ increases the risk of drug resistance. WHO has provided opportunities for the development of alternative antischistosomal drugs. Despite several compounds showing promise against *Schistosoma*, their efficacy was not equivalent to that of PZQ [27, 28]. New therapeutic approaches that combine different modes of action and/or repurposing are therefore urgently required.

Combination chemotherapy is widely exploited in many fields, including the treatment of cancer, bacterial infections, HIV, and malaria, as well as in veterinary medicine [29–33]. In experimental schistosomiasis, promising results have been reported with the use of PZQ in combination with other drugs or biomolecules for multistage targeting, ameliorating infection-associated pathologies, and reducing the risk of drug resistance [12].

In our previous studies, DW-3-15, a PZQ derivative, was developed and proved to have promising activity against schistosomula and adult worms of *S. japonicum* [13, 14]. In this study, we analyzed the effect of a combination of PZQ and DW-3-15 against varying schistosome developmental stages both in vivo and in vitro. Based on the results, PZQ in combination with DW-3-15 improved the antischistosomal effect over any single compound. It showed a potent reduction in vitality and worm burden by combination chemotherapy at 100 μM of the two compounds, respectively, highlighting the utility of the therapy for increasing drug efficacy. Campelo et al. [17] reported that drug combinations, as an alternative approach used in the treatment of several diseases, have shown some advantages in clinical treatment by decreasing long-term toxicity and drug resistance. In our study, halving the concentration/dose of PZQ combined with DW-3-15 maintained a favorable antischistosomal effect. This therapeutic method provides a possibility for reducing drug doses against *S. japonicum*, thereby potentially reducing the side effects and the risk of single-drug resistance. However, whether this therapeutic method actually reduces the side effects of the two compounds remains to be confirmed in future studies.

CI analysis in vitro and in vivo revealed that the groups showed variable degrees of synergy (Figs. 2 and 7). This synergy in antischistosomal activity was likely due to the increased activity of the anthelmintic drugs on different targets [34]. Indeed, there are some differences in the mechanisms of antischistosomal effects between PZQ and DW-3-15. PZQ exerts direct antischistosomal effects on adult worms through its effects on Ca^2+^ homeostasis, resulting in spastic paralysis and rapid vacuolization of the worm surface [35]. The antischistosomal mechanisms of DW-3-15 are less well characterized, but we previously demonstrated its influence on histone acetyltransferase (data not shown). The combination of multi-target compounds of PZQ and DW-3-15 not only improved the therapeutic profile of PZQ, but also potentially reduced the risk of drug resistance.

The tegument of the blood fluke plays a key role in nutrient absorption, proliferation, energy metabolism, secretory functions, and parasite protection against the host immune system [36, 37]. In addition, it is considered to be a major target for antischistosomal drugs [38]. Alterations of the tegument can also be used to evaluate the antischistosomal activity of a compound [39]. Campelo et al. [17] observed extensive soft tissue damage on the surface of *S. mansoni* exposed to combinations of piplartine, epiisopiloturine, and PZQ, and they demonstrated synergistic effects of compound combinations by ultrastructural study.

In this study, ultrastructural alterations in the tegument of adult male schistosomes were observed by SEM. The combination of multi-target compounds increased tegument damage, with extensive peeling, exposure, and injury of the subtegumental muscle layer of *S. japonicum*. The tegumental damage likely increased its exposure to immunogens and immunogenic epitopes [40]. This damage and immunological recognition might represent a key process in the activity of the combination therapy in vivo. Moreover, tegumental changes in the combination group were greater than in those treated with the single compound, with the worms showing significant ultrastructural differences. The results also indicated that DW-3-15 differs from PZQ in terms of its insecticidal mechanism, and the compounds had synergistic effects on the target when used in combination.

Schistosome eggs, when deposited in the host liver or intestinal tissue, are the major cause of pathology in schistosomiasis [4]. They are viable and actively metabolic organisms that are highly antigenic, evoking inflammation that leads to a granulomatous response [4]. Granulomas ultimately lead to liver fibrosis and death in chronically infected hosts [4]. In this study, the combination of PZQ with DW-3-15 significantly attenuated the egg burden in the liver and reduced the area of egg-induced granuloma, indicating that the combination therapy not only possesses antiparasitic activity, but also inhibits the formation of egg-induced granulomas. Thus, compared with single-compound treatment, the combination of PZQ and DW-3-15 could alleviate liver lesions and protect the liver in cases of schistosomiasis.

## Conclusions

In the present study, we confirmed that the combination of PZQ and DW-3-15 produces synergistic effects, and represents a novel potential multistage therapy to increase antischistosomal activity. The combination chemotherapy of PZQ and DW-3-15 can be used against both schistosomula and adult worms, and ameliorates schistosomiasis hepatic pathology. Therefore, this therapy may shed light on new treatments for *S. japonicum* infection. Furthermore, based on our in vitro and in vivo findings, the antischistosomal mechanism of DW-3-15 appears to differ from that of PZQ. When the two compounds are combined, their action targets may be different from those when each is used alone. Further studies are required to fully define the mechanism(s) of antischistosomal action of PZQ and DW-3-15 co-therapy, and to address suitable dosage for the treatment of human schistosomiasis.

## Supplementary Information

**Additional file 1: Table S1**. Viability of *S. japonicum* males exposed to PZQ and DW-3-15 at different combinations of concentrations in vitro.** Table S2**. Viability of *S. japonicum* females exposed to PZQ and DW-3-15 at different combinations of concentrations in vitro.** Table S3**. Viability of *S. japonicum* juveniles exposed to PZQ and DW-3-15 at different combinations of concentrations in vitro.** Table S4**. Antischistosomal effects of *S. japonicum* juvenile (14 days) treated by PZQ and DW-3-15 at different combinations of concentrations in vivo.** Table S5**. Antischistosomal effects of multiple developmental stages of *S. japonicum* treated by PZQ and DW-3-15 at different combinations of concentrations in vivo.** Table S6**. Antischistosomal effects of adult (28 days) of *S. japonicum* treated by PZQ and DW-3-15 at different combinations of concentrations in vivo.

**Additional file 2: Figure S1**. Scanning electron micrographs of *S. japonicum* males exposed to PZQ. After 72 h of incubation with 100 µM PZQ, worms showed obvious damage to the gynecophoral canal (GC) (a). The oral sucker and ventral sucker showed swelling (SW) and loss of spine (S) (b, c). Blisters (BS) (d), pit-shaped erosion (PE) (d, e), shallow sloughing (SL) (f) and disintegration (DI) (f) of the tegumental crest were visible. Scale bars: a, 50 µm; b, 5 µm; c, 5 µm, e, 30 µm, f, 5 µm.

**Additional file 3: Figure S2**. Scanning electron micrographs of *S. japonicum* males exposed to DW-3-15. After 72 h of incubation with 100µM DW-3-15, worms showed severe damage to the gynecophoral canal (GC) (a), the oral sucker and ventral sucker showed obvious hole-shaped erosion (HE) damage and surface fusion with spine loss (b, c); in addition, the tegumental crest displayed disintegration (DI), blisters (BS) and hole-shaped erosion (HE) injury (d, e). Extensive sloughing (SL) with exposure of the subtegumental muscle layer (ML) were observed (f). Scale bars: a, 200 µm; b, 10 µm; c, 5 µm; d, 50 µm; e, 10 µm; f, 30 µm.

**Additional file 4: Figure S3**. Scanning electron micrographs of *S. japonicum* adult males exposed to PD^a^. After 72 h of incubation, the gynecophoral canal (GC) (a) showed severe injury, and the oral sucker and ventral sucker of worms showed swelling (SW) and disintegration (DI) (b, c). The tegumental crest showed hole-shaped erosion (HE), extensive sloughing (SL) with exposure of the subtegumental muscle layer (ML) and muscle injury (MI) (d–f). Scale bars: a, 300 µm; b, 5 µm; c, 10 µm; d, 50 µm; e, 50 µm; f, 30 µm.

**Additional file 5: Figure S4**. Effects on hepatic granulomas of mice treated by PZQ combined with DW-3-15 in vivo. The mice were infected with 14-day-old (a), multiple-stage (b), and 28-day-old worms (c) of *S. japonicum*. The dose of PZQ and DW-3-15 was 200 mg/kg and 400 mg/kg, respectively. PD^c^ was 100 mg/kg PZQ combined with 200 mg/kg DW-3-15; PD^d^ was 200 mg/kg PZQ combined with 400 mg/kg DW-3-15. Significant differences compared to the control group are indicated by *P < 0.05, **P < 0.01 and ***P < 0.001. Scale bars: 200 μm.

## Data Availability

All data generated and analyzed during this study are included within the article and its additional files.

## Abbreviations

PZQ: Praziquantel
WHO: World Health Organization
DALYs: Disability-adjusted life years
DMSO: Dimethyl sulfoxide
PI: Propidium iodide
CI: Combination index
