# Supplementary material for: Synergistic effect of combination chemotherapy with praziquantel and DW-3-15 for Schistosoma japonicum in vitro and in vivo
Source: Parasit Vectors. 2021 Oct 26;14:550. doi: 10.1186/s13071-021-05065-x (PMC8549225; doi:10.1186/s13071-021-05065-x)
Supplement: Supplementary file 1 — Additional file 1: Table S1. Viability of S. japonicum males exposed to PZQ and DW-3-15 at different combinations of concentrations in vitro. Table S2. Viability of S. japonicum females exposed to PZQ and DW-3-15 at different combinations of concentrations in vitro. Table S3. Viability of S. japonicum juveniles exposed to PZQ and DW-3-15 at different combinations of concentrations in vitro. Table S4. Antischistosomal effects of S. japonicum juvenile (14 days) treated by PZQ and DW-3-15 at different combinations of concentrations in vivo. Table S5. Antischistosomal effects of multiple developmental stages of S. japonicum treated by PZQ and DW-3-15 at different combinations of concentrations in vivo. Table S6. Antischistosomal effects of adult (28 days) of S. japonicum treated by PZQ and DW-3-15 at different combinations of concentrations in vivo. [file 13071_2021_5065_MOESM1_ESM.pdf]

Table S1 Viability of *S. japonicum* males exposed to PZQ and DW-3-15 at different combinations of concentrations *in vitro*

Compound	Concentration (μM)	24 h		48 h		72 h	
		Worm survival rate (%)	Viability score (Mean ± SEM)/Viability reduction rate (%)	Worm survival rate (%)	Viability score (Mean ± SEM)/Viability reduction rate (%)	Worm survival rate(%)	Viability score (Mean ± SEM)/Viability reduction rate (%)
Control[#]	-	100.00	3.00 ± 0.00/0.00	100.00	3.00 ± 0.00/0.00	100.00	3.00 ± 0.00/0.00
PZQ	25	100.00	1.50 ± 0.11/50.00[****]	100.00	1.93 ± 0.12/35.56[****]	100.00	2.13 ± 0.13/28.89[****]
	50	80.00	1.03 ± 0.12/65.56[****]	96.67	1.60 ± 0.11/46.67[****]	96.67	1.67 ± 0.14/44.44[****]
	75	70.00	0.83 ± 0.12/72.22[****]	80.00	1.00 ± 0.12/66.67[****]	96.67	1.13 ± 0.08/62.22[****]
	100	43.33	0.43 ± 0.09/85.56[****]	76.67	0.90 ± 0.11/70.00[****]	86.67	0.97 ± 0.09/67.78[****]
DW-3-15	25	100.00	2.70 ± 0.10/10.00	93.33	2.40 ± 0.17/20.00[***]	86.67	2.30 ± 0.19/23.33[****]
	50	66.67	0.80 ± 0.13/73.33[****]	53.33	0.57 ± 0.10/81.11[****]	50.00	0.50 ± 0.09/83.33[****]
	75	50.00	0.50 ± 0.09/83.33[****]	30.00	0.30 ± 0.09/90.00[****]	26.67	0.30 ± 0.10/90.00[****]
	100	20.00	0.20 ± 0.07/93.33[****]	16.67	0.17 ± 0.07/94.44[****]	16.67	0.17 ± 0.07/94.44[****]
PD[a]	50 + 50	13.33	0.13 ± 0.06/95.56[****]	33.33	0.33 ± 0.09/88.89[****]	16.67	0.17 ± 0.07/94.44[****]
PD[b]	100 + 100	0.00	0.00 ± 0.00/100.00[****]	6.67	0.07 ± 0.05/97.78[****]	0.00	0.00 ± 0.00/100.00[****]

The worms were exposed to the chemicals for 16 h, rinsed three times with DMEM the next day, then cultured in chemical-free complete DMEM, worm viability were observed at 24, 48 and 72 h post-incubation; [#]The control group was incubated with complete DMEM with 0.1% DMSO; PD[a] means the combination of 50 μM PZQ with 50 μM DW-3-15; PD[b] means the combination of 100 μM PZQ with 100 μM DW-3-15; Significant differences compared to the control group are indicated by [***] $P < 0.001$, [****] $P < 0.0001$

Table S2 Viability of *S. japonicum* females exposed to PZQ and DW-3-15 at different combinations of concentrations *in vitro*

Compound	Concentration (μM)	24 h		48 h		72 h	
		Worm survival rate (%)	Viability score (Mean ± SEM)/Viability reduction rate (%)	Worm survival rate (%)	Viability score (Mean ± SEM)/Viability reduction rate (%)	Worm survival rate (%)	Viability score (Mean ± SEM)/Viability reduction rate (%)
Control[#]	-	100.00	3.00 ± 0.00/0.00	100.00	3.00 ± 0.00/0.00	100.00	3.00 ± 0.00/0.00
PZQ	25	96.67	1.23 ± 0.09/59.00****	93.33	1.67 ± 0.11/44.33****	100.0	1.77 ± 0.09/41.00****
	50	73.33	0.97 ± 0.13/67.67****	80.00	1.07 ± 0.13/64.33****	96.67	1.17 ± 0.08/61.00****
	75	63.33	0.80 ± 0.13/73.33****	80.00	1.00 ± 0.12/66.67****	90.00	1.07 ± 0.10/64.33****
	100	56.67	0.67 ± 0.12/77.67****	73.33	0.77 ± 0.09/74.33****	83.33	0.83 ± 0.07/72.33****
DW-3-15	25	93.33	2.00 ± 0.17/33.33****	93.33	1.93 ± 0.17/35.67****	83.33	1.90 ± 0.21/36.67****
	50	93.33	0.93 ± 0.05/69.00****	86.67	0.87 ± 0.06/71.00****	53.33	0.53 ± 0.09/82.33****
	75	60.00	0.60 ± 0.09/80.00****	33.33	0.33 ± 0.09/89.00****	26.67	0.27 ± 0.08/91.00****
	100	33.33	0.33 ± 0.09/89.00****	20.00	0.20 ± 0.07/93.33****	16.67	0.17 ± 0.07/94.33****
PD[a]	50 + 50	30.00	0.30 ± 0.10/90.00****	33.33	0.33 ± 0.09/89.00****	16.67	0.20 ± 0.09/93.33****
PD[b]	100 + 100	26.67	0.27 ± 0.08/91.00****	20.00	0.20 ± 0.07/93.33****	3.33	0.03 ± 0.03/99.00****

The worms were exposed to the chemicals for 16 h, rinsed three times with DMEM the next day, then cultured in chemical-free complete DMEM, worm viability were observed at 24, 48 and 72 h post-incubation; [#]The control group was incubated with complete DMEM with 0.1% DMSO; PD[a] means the combination of 50 μM PZQ with 50 μM DW-3-15; PD[b] means the combination of 100 μM PZQ with 100 μM DW-3-15; Significant differences compared to the control group are indicated by **** $P < 0.0001$

Table S3 Viability of *S. japonicum* juveniles exposed to PZQ and DW-3-15 at different combinations of concentrations *in vitro*

Compound	Concentration (μM)	24 h		48 h		72 h	
		Worm survival rate (%)	Viability score (Mean ± SEM)/Viability reduction rate (%)	Worm survival rate (%)	Viability score (Mean ± SEM)/Viability reduction rate (%)	Worm survival rate (%)	Viability score (Mean ± SEM)/Viability reduction rate (%)
Control[#]	-	100.00	3.00 ± 0.00/0.00	100.00	3.00 ± 0.00/0.00	100.00	3.00 ± 0.00/0.00
PZQ	25	76.67	1.63 ± 0.21/45.56****	93.33	1.70 ± 0.17/43.33****	93.33	1.80 ± 0.17/40.00****
	50	66.67	1.13 ± 0.19/62.22****	76.67	1.27 ± 0.17/57.78****	86.67	1.50 ± 0.18/50.00****
	75	46.67	0.77 ± 0.18/74.44****	63.33	0.97 ± 0.17/67.78****	70.00	1.17 ± 0.18/61.11****
	100	43.33	0.53 ± 0.13/82.22****	56.67	0.73 ± 0.14/75.56****	70.00	0.93 ± 0.15/68.89****
DW-3-15	25	60.00	1.03 ± 0.19/65.56****	43.33	0.60 ± 0.15/80.00****	40.00	0.60 ± 0.17/80.00****
	50	46.67	0.70 ± 0.16/76.67****	23.33	0.43 ± 0.16/85.56****	23.33	0.37 ± 0.15/87.78****
	75	30.00	0.30 ± 0.09/90.00****	16.67	0.17 ± 0.07/94.44****	13.33	0.13 ± 0.06/95.56****
	100	20.00	0.27 ± 0.11/91.11****	13.33	0.13 ± 0.06/95.56****	6.67	0.07 ± 0.05/97.78****
PD[a]	50 + 50	26.67	0.27 ± 0.11/91.11****	0.00	0.00 ± 0.00/100.00****	0.00	0.00 ± 0.00/100.00****
PD[b]	100 + 100	13.33	0.13 ± 0.06/95.56****	0.00	0.00 ± 0.00/100.00****	0.00	0.00 ± 0.00/100.00****

The worms were exposed to the chemicals for 16 h, rinsed three times with DMEM the next day, then cultured in chemical-free complete DMEM, worm viability were observed at 24, 48 and 72 h post-incubation; [#]The control group was incubated with complete DMEM with 0.1% DMSO; PD[a] means the combination of 50 μM PZQ with 50 μM DW-3-15; PD[b] means the combination of 100 μM PZQ with 100 μM DW-3-15; Significant differences compared to the control group are indicated by **** $P < 0.0001$

Table S4 Antischistosomal effects of *S. japonicum* juvenile (14 d) treated by PZQ and DW-3-15 at different combinations of concentrations *in vivo*

Compound	Dose (mg/kg)	Worm burden (Mean ± SEM)			Worm burden reduction (%)			Eggs burden (Mean ± SEM)	Egg reduction (%)
		Female	Male	Total	Female	Male	Total		
Control#	-	26.2 ± 0.6	30.1 ± 0.5	56.3 ± 0.8	-	-	-	106400.0 ± 5503.9	-
PZQ	100	20.0 ± 0.9****	27.3 ± 0.9	47.3 ± 0.8****	23.7	9.3	16.0	59005.4 ± 3841.4****	44.5
	200	17.1 ± 1.2****	23.4 ± 1.0****	40.5 ± 1.4****	34.7	22.3	28.1	22829.8 ± 990.3****	78.5
	400	13.5 ± 1.0****	18.4 ± 0.9****	31.9 ± 1.7****	48.5	38.9	43.3	17826.2 ± 1474.4****	83.2
DW-3-15	100	14.3 ± 0.8****	20.3 ± 1.3****	34.7 ± 1.6****	45.4	32.6	38.4	24712.8 ± 1718.1****	76.8
	200	12.7 ± 0.8****	15.5 ± 0.9****	28.1 ± 1.6****	51.5	48.5	50.1	20040.6 ± 1072.5****	81.2
	400	6.7 ± 0.6****	10.0 ± 0.6****	16.7 ± 1.0****	74.4	66.8	70.3	16330.5 ± 890.5****	84.7
PD[c]	100 + 200	7.3 ± 0.7****	11.0 ± 1.1****	18.3 ± 1.7****	72.1	63.5	67.5	3757.5 ± 501.1****	96.5
PD[d]	200 + 400	3.1 ± 0.5****	6.1 ± 0.9****	9.1 ± 1.3****	88.2	79.7	83.8	376.4 ± 144.1****	99.6

#The control group was treated with 0.5% carboxymethyl cellulose sodium; PD[c] means 100 mg/kg PZQ combined with 200 mg/kg DW-3-15; PD[d] means 200 mg/kg PZQ combined with 400 mg/kg DW-3-15; Significant differences compared to the control group are indicated by **** $P < 0.0001$

Table S5 Antischistosomal effects of multiple developmental stages of *S. japonicum* treated by PZQ and DW-3-15 at different combinations of concentrations *in vivo*

Compound	Dose (mg/kg)	Worm burden (Mean ± SEM)			Worm burden reduction (%)			Eggs burden (Mean ± SEM)	Egg reduction (%)
		Female	Male	Total	Female	Male	Total		
Control[#]	-	24.3 ± 1.1	31.7 ± 0.6	56.0 ± 0.7	-	-	-	106400.0 ± 5503.9	-
PZQ	100	14.9 ± 1.1****	22.5 ± 1.3****	37.4 ± 2.1****	38.7	29.0	33.2	41440.0 ± 2139.3****	61.1
	200	13.0 ± 0.7****	16.8 ± 1.2****	29.8 ± 1.6****	46.5	47.0	46.8	38800.0 ± 3375.7****	63.5
	400	8.2 ± 0.9****	11.9 ± 1.0****	20.1 ± 1.8****	66.3	62.5	64.1	21920.0 ± 2160.5****	79.4
DW-3-15	100	11.3 ± 0.4****	16.4 ± 0.9****	27.7 ± 1.0****	53.5	48.3	50.5	49600.0 ± 1878.1****	53.4
	200	11.0 ± 0.8****	15.2 ± 1.1****	26.2 ± 1.5****	54.7	52.1	53.2	35333.3 ± 2317.4****	66.8
	400	5.7 ± 0.4****	8.7 ± 0.4****	14.3 ± 0.7****	76.5	72.6	74.5	27120.0 ± 1183.3****	74.5
PD[c]	100 + 200	7.8 ± 0.8****	11.3 ± 1.0****	19.1 ± 1.6****	67.9	64.4	65.9	14880.0 ± 1817.3****	86.0
PD[d]	200 + 400	2.9 ± 0.4****	6.7 ± 0.9****	9.5 ± 1.2****	88.1	78.9	83.0	6240.0 ± 874.7****	94.1

[#]The control group was treated with 0.5% carboxymethyl cellulose sodium; PD[c] means 100 mg/kg PZQ combined with 200 mg/kg DW-3-15; PD[d] means 200 mg/kg PZQ combined with 400 mg/kg DW-3-15; Significant differences compared to the control group are indicated by **** $P < 0.0001$

Table S6 Antischistosomal effects of adult (28 d) of *S. japonicum* treated by PZQ and DW-3-15 at different combinations of concentrations *in vivo*

Compound	Dose (mg/kg)	Worm burden (Mean ± SEM)			Worm burden reduction (%)			Egg burden (Mean ± SEM)	Egg reduction (%)
		Female	Male	Total	Female	Male	Total		
Control[#]	-	24.6 ± 0.6	30.1 ± 0.5	54.7 ± 0.6	-	-	-	106400.0 ± 5503.9	-
PZQ	100	2.9 ± 0.5****	8.4 ± 1.5****	11.3 ± 1.9****	88.2	72.1	79.3	37600.0 ± 2913.9****	64.7
	200	1.6 ± 0.5****	4.3 ± 0.7****	5.9 ± 1.1****	93.5	85.7	89.2	23280.1 ± 1045.6****	78.1
	400	0.5 ± 0.1****	1.2 ± 0.2****	1.7 ± 0.3****	98.0	96.0	96.9	15750.1 ± 1174.0****	85.2
DW-3-15	100	10.7 ± 1.0****	20.5 ± 1.1****	31.1 ± 1.7****	56.5	31.9	43.1	46720.0 ± 2799.9****	56.1
	200	8.0 ± 1.0****	18.1 ± 1.2****	26.1 ± 1.7****	67.5	39.9	52.3	29457.3 ± 770.7****	72.3
	400	7.2 ± 0.7****	14.7 ± 0.8****	21.9 ± 1.1****	70.7	51.2	60.0	23418.2 ± 1871.8****	78.0
PD[c]	100 + 200	1.2 ± 0.4****	3.1 ± 0.8****	4.3 ± 1.1****	95.1	89.7	92.1	19840.0 ± 1098.2****	81.4
PD[d]	200 + 400	0.1 ± 0.1****	1.3 ± 0.4****	1.5 ± 0.4****	99.6	95.7	97.3	13468.1 ± 864.6****	87.3

[#]The control group was treated with 0.5% carboxymethyl cellulose sodium; PD[c] means 100 mg/kg PZQ combined with 200 mg/kg DW-3-15; PD[d] means 200 mg/kg PZQ combined with 400 mg/kg DW-3-15; Significant differences compared to the control group are indicated by **** $P < 0.0001$
