# Supplementary figures and images for: Synergistic effect of combination chemotherapy with praziquantel and DW-3-15 for Schistosoma japonicum in vitro and in vivo
Source: Parasit Vectors. 2021 Oct 26;14:550. doi: 10.1186/s13071-021-05065-x (PMC8549225; doi:10.1186/s13071-021-05065-x)

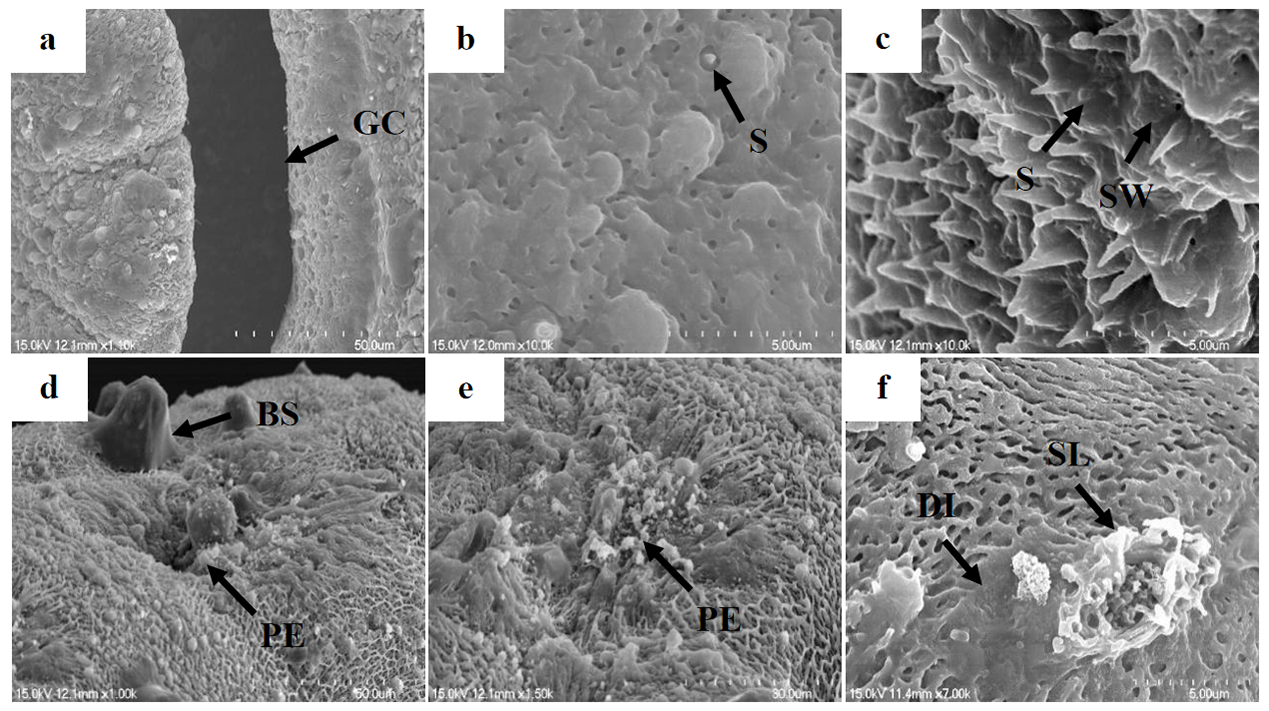

Supplement: Supplementary file 2 — Additional file 2: Figure S1. Scanning electron micrographs of S. japonicum males exposed to PZQ. After 72 h of incubation with 100 µM PZQ, worms showed obvious damage to the gynecophoral canal (GC) (a). The oral sucker and ventral sucker showed swelling (SW) and loss of spine (S) (b, c). Blisters (BS) (d), pit-shaped erosion (PE) (d, e), shallow sloughing (SL) (f) and disintegration (DI) (f) of the tegumental crest were visible. Scale bars: a, 50 µm; b, 5 µm; c, 5 µm, e, 30 µm, f, 5 µm. [file 13071_2021_5065_MOESM2_ESM.tif]

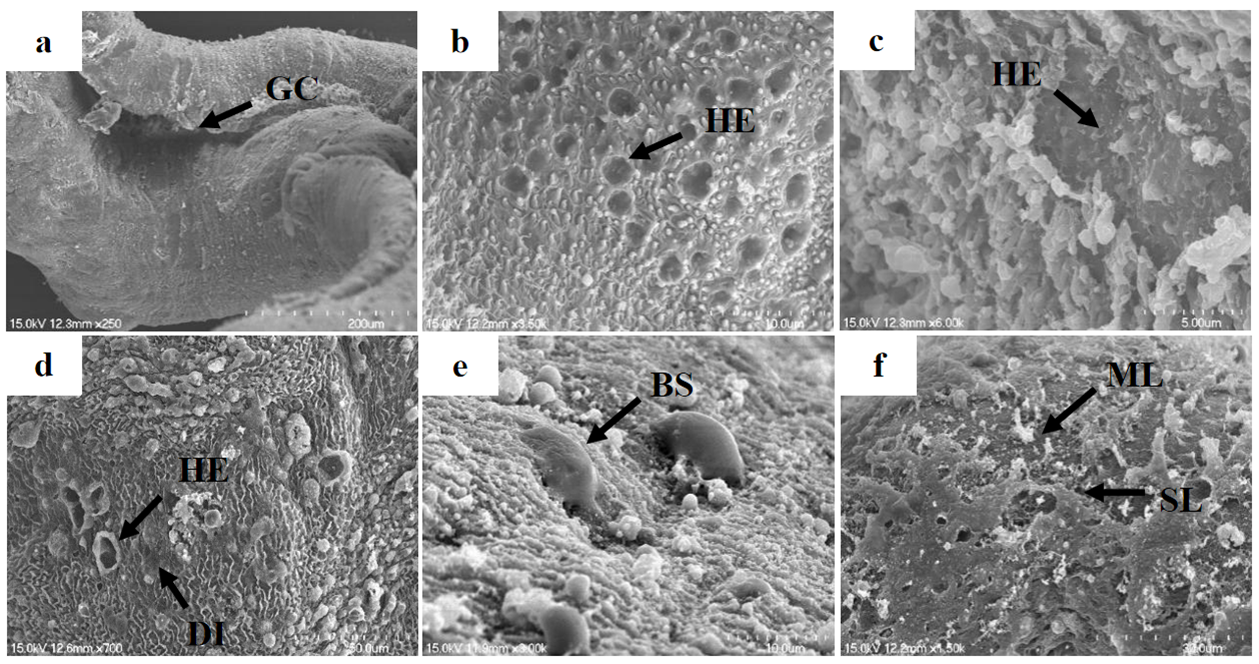

Supplement: Supplementary file 3 — Additional file 3: Figure S2. Scanning electron micrographs of S. japonicum males exposed to DW-3-15. After 72 h of incubation with 100µM DW-3-15, worms showed severe damage to the gynecophoral canal (GC) (a), the oral sucker and ventral sucker showed obvious hole-shaped erosion (HE) damage and surface fusion with spine loss (b, c); in addition, the tegumental crest displayed disintegration (DI), blisters (BS) and hole-shaped erosion (HE) injury (d, e). Extensive sloughing (SL) with exposure of the subtegumental muscle layer (ML) were observed (f). Scale bars: a, 200 µm; b, 10 µm; c, 5 µm; d, 50 µm; e, 10 µm; f, 30 µm. [file 13071_2021_5065_MOESM3_ESM.tif]

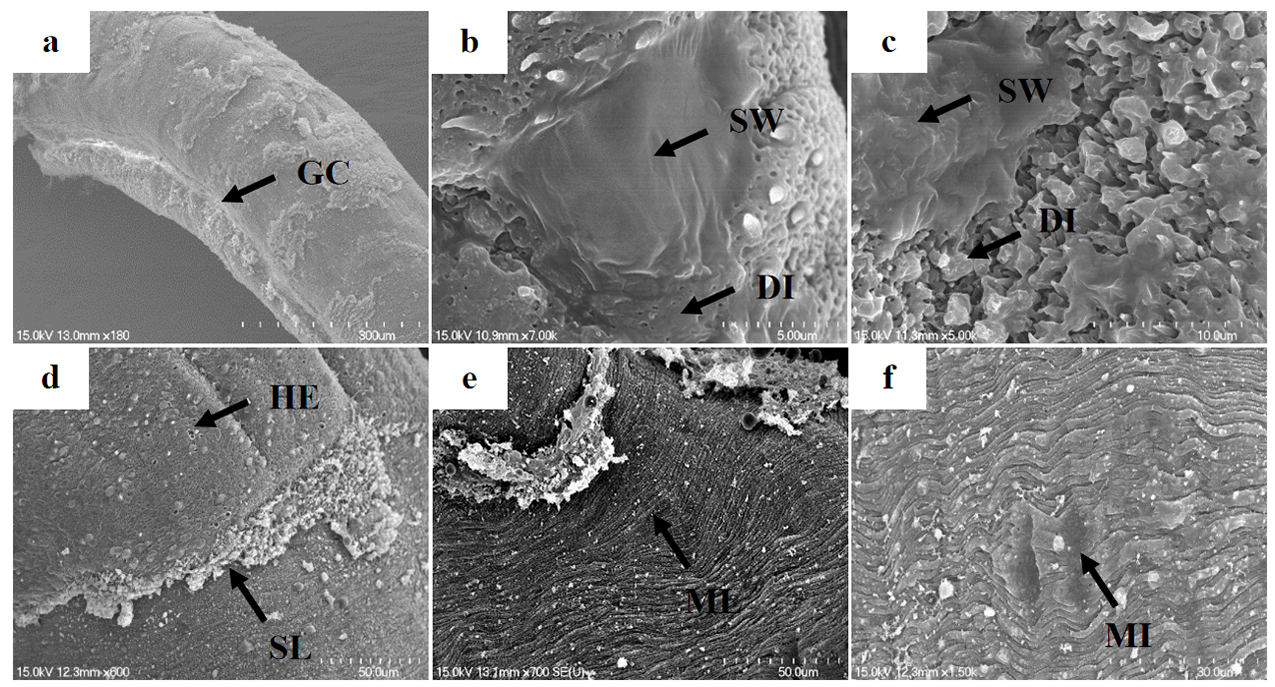

Supplement: Supplementary file 4 — Additional file 4: Figure S3. Scanning electron micrographs of S. japonicum adult males exposed to PDa. After 72 h of incubation, the gynecophoral canal (GC) (a) showed severe injury, and the oral sucker and ventral sucker of worms showed swelling (SW) and disintegration (DI) (b, c). The tegumental crest showed hole-shaped erosion (HE), extensive sloughing (SL) with exposure of the subtegumental muscle layer (ML) and muscle injury (MI) (d–f). Scale bars: a, 300 µm; b, 5 µm; c, 10 µm; d, 50 µm; e, 50 µm; f, 30 µm. [file 13071_2021_5065_MOESM4_ESM.tif]

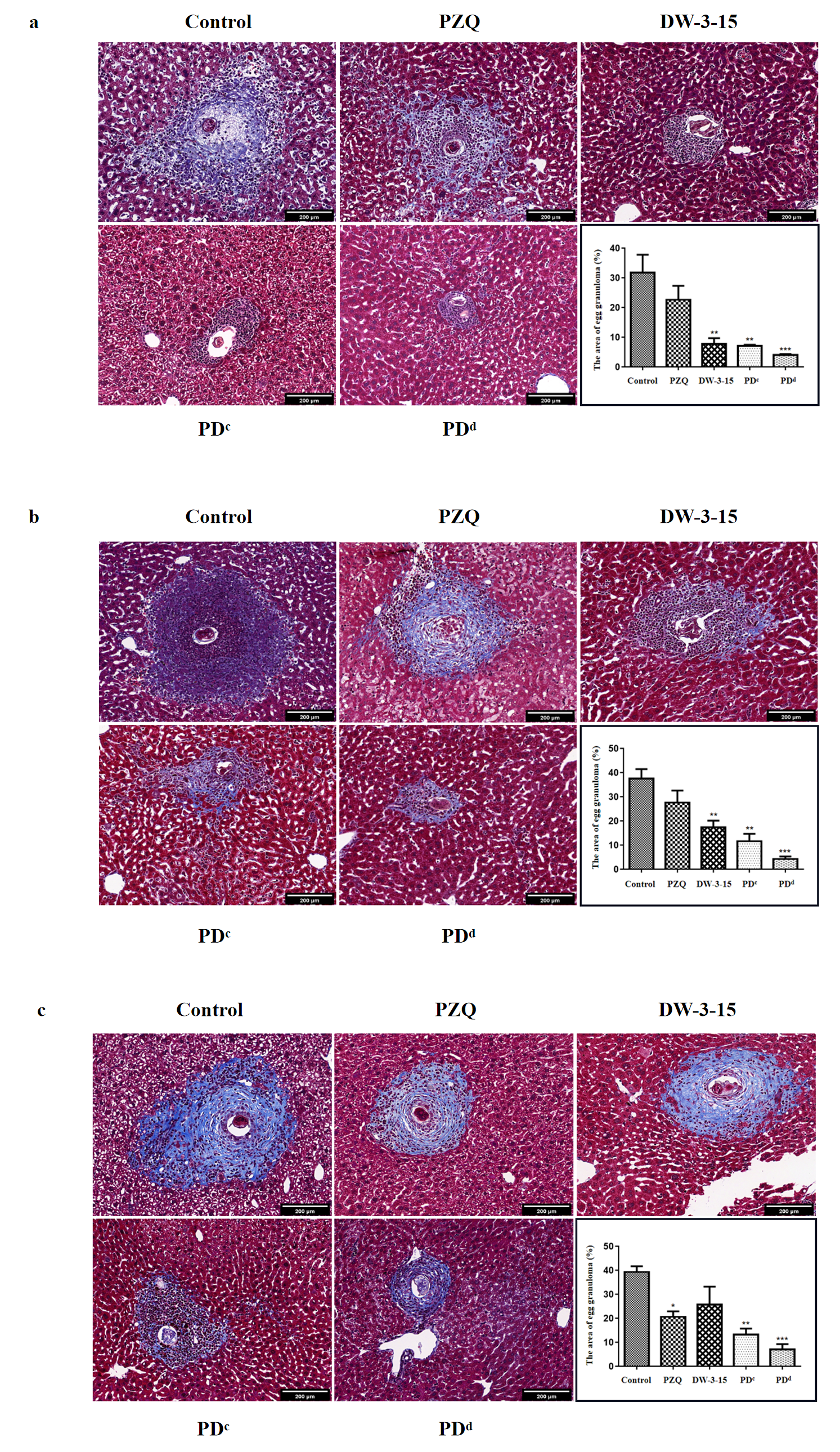

Supplement: Supplementary file 5 — Additional file 5: Figure S4. Effects on hepatic granulomas of mice treated by PZQ combined with DW-3-15 in vivo. The mice were infected with 14-day-old (a), multiple-stage (b), and 28-day-old worms (c) of S. japonicum. The dose of PZQ and DW-3-15 was 200 mg/kg and 400 mg/kg, respectively. PDc was 100 mg/kg PZQ combined with 200 mg/kg DW-3-15; PDd was 200 mg/kg PZQ combined with 400 mg/kg DW-3-15. Significant differences compared to the control group are indicated by *P < 0.05, **P < 0.01 and ***P < 0.001. Scale bars: 200 μm. [file 13071_2021_5065_MOESM5_ESM.tif]
